# Transcriptomic analysis of midbrain and individual hindbrain rhombomeres in the chick embryo

**DOI:** 10.1038/sdata.2014.14

**Published:** 2014-07-22

**Authors:** Leigh Wilson, David Chambers

**Affiliations:** 1 MRC Centre for Developmental Neurobiology, King’s College London, 4th Floor New Hunt’s House, London SE1 1UL, UK; 2 Drug Discovery Unit, Wolfson Centre for Age Related Disease, King’s College London, Guy’s Campus, London SE1 1UL, UK

## Abstract

The anteroposterior compartments of the developing hindbrain (rhombomeres [r]) are normally patterned by the combinatorial action of distinct Hox genes. Using Affymetrix GeneChips to define the repertoire of genes regulated in each rhombomere, we have performed a systematic survey of the transcriptional status of individual segments of the developing chick hindbrain (r1-5) at a key stage of early development (HH11) and identified hundreds of previously un-described genes expressed in this region. For comparative purposes, we have also included the adjacent region of the embryonic midbrain (m) in our dataset. In summary, six different embryonic brain regions (m, r1, r2, r3, r4 & r5) are represented by biological duplicates to give a raw dataset comprised of 12 individual Affymetrix GeneChip Cel and CHP files. These data give an opportunity to assess the genome-wide complexity of gene expression during patterning of the chick developing midbrain and hindbrain, and may be relevant to extending our understanding of the genes regulated by Hox family transcription factors.

## Background & Summary

The vertebrate central nervous system (CNS) consists of thousands of distinct neuronal cells, each with unique molecular genetic profiles, organised into complex patterns of circuitry that ensure appropriate functional output. A key goal of modern developmental neuroscience is to unravel the molecular logic that first establishes the diversity in neuronal cell type and subsequently dictates their development into integrated higher order networks. Recent years have seen considerable progress in establishing the initial framework that underpins the acquisition of neuronal identity. Several lines of evidence have shown that cells are specified in discrete steps within the neuroepithelium influenced by the anteroposterior (AP) and dorsoventral (DV) position of the precursor cell. In response to diffusible molecular cues released by discrete cell groups known as local organisers a series of high level ‘selector’ genes establish the first molecular co-ordinates that imprint progenitor cells with an outline fate^1–3^. However, despite this being a well-recognised and investigated paradigm, we still remain relatively ignorant of the repertoire of subsequent molecular ‘effectors’ that then impart specific facets of neuronal phenotype, behaviour and connectivity. The developing vertebrate hindbrain and the adjacent mid-hindbrain boundary (MHB) signalling centre provide the ideal accessible systems in which to examine the molecular controls underpinning the emergence of neuronal diversity^4–7^.

The Hox family of homeodomain transcription factors are key determinants of cell specification and identity during animal development^8,9^. However, despite their well-defined roles in the establishment of anteroposterior (AP) pattern and considerable research into their mechanism of action, relatively few target genes have been identified in the downstream regulatory network (e.g., ref. 10). We have sought to investigate this issue, focusing on the developing hindbrain and the cranial motor neurons that arise from this region. The reiterated cell lineage-restricted anteroposterior compartments of the developing hindbrain (rhombomeres (r)) are normally patterned by the combinatorial action of distinct Hox genes^7,11–14^.

Exploiting the relationship between early anatomical distinctions and key selector gene activities of these territories we have generated highly representative and validated genome-wide expression libraries for each division of the hindbrain (rhombomeres) and the caudal portion of the midbrain. Our dataset comprises systematic surveys of the transcriptional status of individual segments of the developing chick hindbrain (r1-5) and the adjacent region of the embryonic midbrain (m) during the HH11 stage of chick development. In summary, six different embryonic brain regions (m, r1, r2, r3, r4 & r5) are represented by biological duplicates to give a raw dataset comprised of 12 individual Affymetrix GeneChip Cel and CHP files.

These data can be exploited to investigate how neuronal specificity is achieved by the recruitment of effector genes and will afford insights into 3 key areas;

- What and how are the developmental genetic factors used in response the patterning influences of the mid-hindbrain signalling centre?
- For the understanding of how Hox genes and signalling molecules drive downstream networks of effector gene activity
- Driving the differentiation of embryonic-derived stem cells towards specific fates

A more complete understanding of the molecular logic of neuronal specification and how patterning instructions are relayed to a defined set of cellular cues is a prerequisite to expanding out knowledge of nervous system function in health and disease and for developing rational approaches to progressing stem cells towards a defined fate for use in restorative medicine.

This study complements our previous approaches to address similar questions in the chick and mouse developing midbrain and hindbrain^4–6^. These mouse and chick parallel datasets can be used to define the underlying conserved genetic modules controlling the equivalent patterning processes in the midbrain and hindbrain as well as serving to cross validate each other. Similarly, these data will offer insights into the molecular mechanism that underpin the fundamental patterning differences between mouse and chick. For example, the migration of cell bodies of the r4-derived facial motor nucleus differs between the two species and the molecular genetic control of this is likely to be reflected in the array data sets.

## Methods

### Developing brain tissue collection

**Figure 1:**
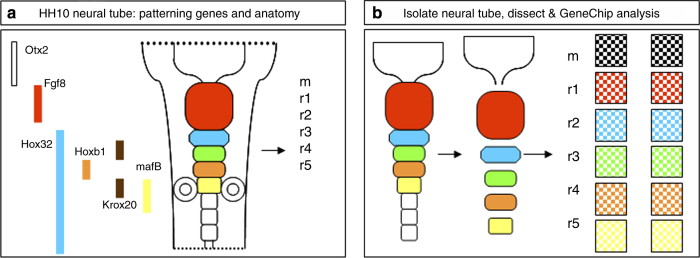
Schematic diagram of neural tube, the key patterning genes and outline of experimental strategy. (**a**) Schematic diagram of the mid-hindbrain region at chick HH10 and some of the key patterning genes expressed at that time. (**b**) To assess the suite of genes expressed in each individual rhombomere or the caudal hindbrain, neural tube tissue was isolated from other surrounding tissues, dissected into the appropriate regions and the resulting RNA analysed by profiling on Affymetrix Chicken GeneChip arrays.

Neural tubes from stage-matched embryonic day (E) 3 [HH11] chick embryos were isolated from surrounding ectoderm and mesoderm as follows; embryos were removed from the egg, washed in phosphate buffered saline (PBS) for 5 min and then transferred to 1 mg/ml Dispase for 5 min. Mechanical dissection with sharpened tungsten needles was used to isolate the entire neural tube free of mesoderm and ectoderm. The characteristic neuromeric landmarks of the developing neural tube were used to guide the dissection (Figure 1a). Neural tubes were returned to PBS and divided into the appropriate midbrain (m) and rhombomere (r1-5) sections (Figure 1b).

### RNA extraction

Total RNA was isolated from each pool using the Absolutely RNA microprep kit in conjunction with DNase-treatment (Agilent Technologies). Tissue samples collected by the above criteria but on different occasions by the same operator were designated as biological replicates. Thus, each region of the neural tube is represented by duplicate pools (denoted as sets 1–2).

### RNA labelling & oligonucleotide array

Labelled extracts, for processing on microarrays, were generated from 10 ng of total RNA by the NuGen Ovation V2 protocol (NuGEN Technologies Inc). Labelled extracts (7 μg SPIA-generated cDNA) for each sample were hybridised to Affymetrix Chicken GeneChips at 45 °C for 20 h before being washed, stained (GeneChip® Fluidics Station 450) and scanned (GeneChip Scanner 3000 7G) as per manufacturer’s instructions (NuGen Technologies Inc & Affymetrix) to produce DAT files, and as described in ref 3.

### Probe-level intensity estimation

The DAT file contains generated from the Affymetrix scanner contain multiple pixel intensity values for each probe on the GeneChip. The DAT files are processed by the Affymetrix image analysis package (MAS, GCOS or AGCC) into a Cel file format that represents a single intensity value (i.e., the multiple pixel intensity values are collapsed into a single value) for each probe on the array (Table 1). For this Affymetrix data format, the Cel files comprise signal intensity values for both the perfect match (PM) probes and the mismatch (MM) probes. The MM probe sets differ from the PM by a single base at position 13 in the probe sequence (25-mer) and are designed to give a quantitative measure of ‘non specific’ binding to the PM. Depending of the type of further bioinformatic analysis chosen, each PM intensity value may be ‘adjusted’ by the cognate MM value (e.g., MAS5 & GC-RMA pre-processing) or left unaltered (e.g., RMA pre-processing).

### Gene-level expression

For the Affymetrix Chicken GeneChip used here, each gene sequence is represented by 11 independent probes on an array. The final expression value for a transcript is derived from the Cel file by summarisation. This is a process where the 11 probe set intensity values are transformed into a single value that is used to represent the expression level. The data files described here have been summarised by the Affymetrix MAS5 algorithm and represented in the CHP (txt) files. These txt files therefore contain information on each probe set identity, a single expression value and some biological descriptors of the gene identity and function. In addition, Affymetrix associate a ‘flag’ designation for each gene expression value that represents measure of the signal reliability; P (Present). M (Marginal) & A (Absent). Thus, the MAS5 processed expression values have been adjusted for the signals detected by the MM probes (Table 2). However, MAS5 pre-processing of the Cel files is just one of a number of algorithms that can be used to summarise the data. Other options for Cel summarisation include the RMA, GC-RMA and PLIER algorithms. Each of these processes transforms the Cel file data in a different way and results in unique profile of gene expression values following summarisation. The choice of a Cel file processing algorithm is user defined.

## Data Records

A raw dataset, comprised of 12 individual Affymetrix GeneChip Cel and CHP files, representing biological duplicates of six different embryonic brain regions (m, r1, r2, r3, r4 & r5, Set 1 & 2) is deposited in the Gene Expression Omnibus (Data Citation 1).

## Technical Validation

### RNA extraction

**Figure 2:**
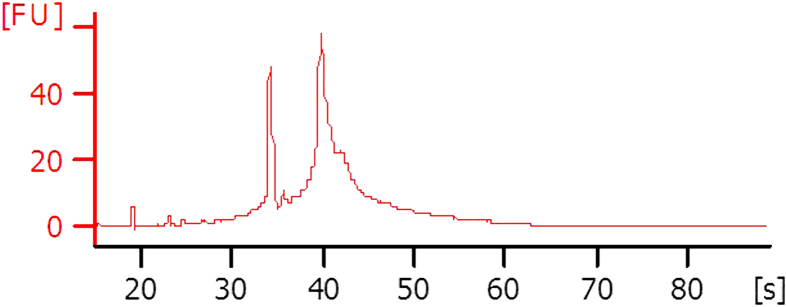
Bioanalyzer analysis of total RNA integrity. Representative electropherogram of Bioanalyzer analysis of total RNA integrity from an individual hindbrain region. Prior to further processing and labelling for microarray hybridisation, all total RNAs were checked on mRNA Pico chips and found to have a RNA Integrity Number (RIN) >7.

Prior to further processing, the integrity of the RNA was monitored on a Bioanalyzer *via* mRNA Pico chips. Total RNA populations derived from each neural tube segment each recorded RNA Integrity numbers greater than 7 (Figure 2; Agilent Technologies Ltd).

### RNA amplification & labelling: NuGen ovation V2 & biotin module

**Figure 3:**
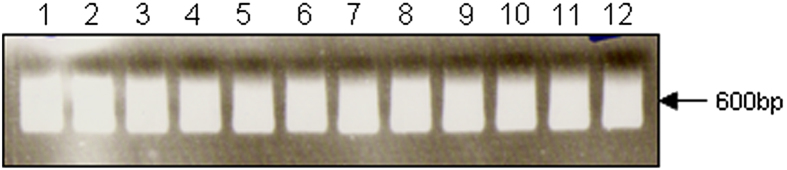
NuGen Ovation V2 SPIA amplifications of m-r5 set 1 & 2. Lanes 1-12. Aliquots of m-r5 set 1 & m-r5 set 2 NuGen Ovation V2 SPIA amplifications were analysed by both agarose gel electrophoresis and spectrophotometry (not shown). The yield of the SPIA amplification reactions was >7 μg from 10 ng total RNA in all samples. The upper size limit of around 1-2 kb, with the majority ‘smear’ of the amplified cDNA below 600 bp and equivalent yields, is a good indicator of reaction success.

Aliquots of m-r5 set 1 & m-r5 set 2 NuGen Ovation V2 SPIA amplifications were analysed by both agarose gel electrophoresis (Figure 3) and spectrophotometry. The yield of the SPIA amplification reactions was >7 μg from 10 ng total RNA in all samples. The analysis of 1 μl of the SPIA reaction volume (i.e., amplified cDNA; 35 μl total final volume) was visualized on an agarose gel. The upper size limit of around 1-2 kb, with the majority ‘smear’ of the amplified cDNA below 600 bp and equivalent yields, indicated reaction efficacy. To minimize potential variation introduced by batch effects, all samples were processed in parallel using reagents assembled from a master mix (sufficient for all 12 samples). Therefore, the main source of variation in the dataset will be from either collection of the biological replicates or the inherent variation between the different brain regions under study.

### GeneChip hybridization

Following application of the labeled extracts to Chicken Genome GeneChips the efficacy of the hybridisation was monitored across the dataset using the following criteria: Average Background=28–34, Raw Q=0.6–0.82 and %*P*=26–45. In each case, all QC metrics were within the recommended manufacturers’ range.

### Proof of concept

**Figure 4:**
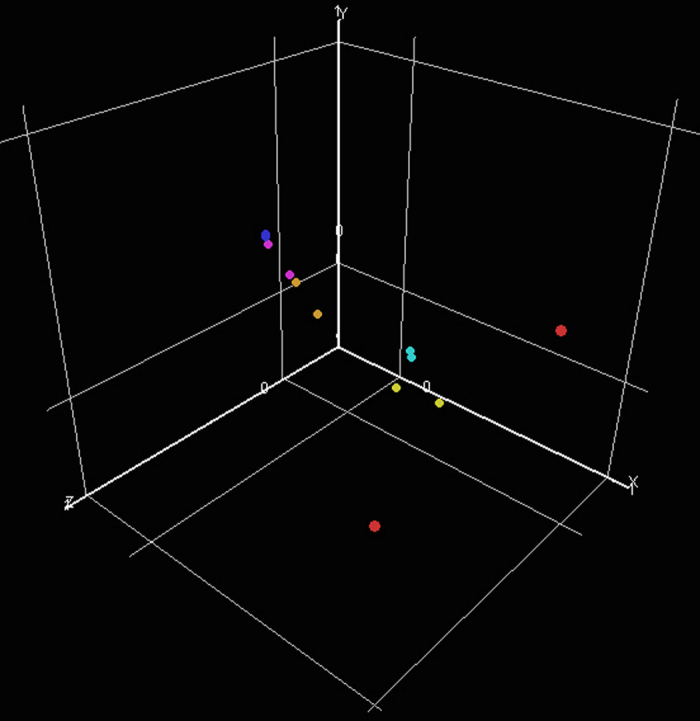
Principle components analysis of individual experimental samples. The relationship within and between biological replicates (m—r5, sets 1-2) was investigated using PCA. Following normalisation and the removal of non expressed genes, the overall distribution of gene expression levels each sample was checked with PCA (PCA 1=38.3%, PCA 2=12.7%, PCA 3=9.647%). Biological replicates are represented by similarly coloured dots (m=blue, r1=cyan, r2=orange, r3=red, r4=yellow, r5=turquoise). The proximity of the similarly coloured dots to each other reveal that the biological replicates are more related to each other than any other sample.

**Figure 5:**
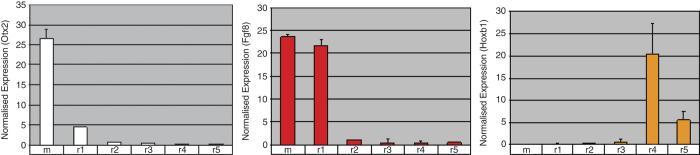
Proof of concept: microarray expression profiles of key mid-hindbrain patterning genes. Normalised microarray expression profiles of Otx2, Fgf8 and Hoxb1 reveals significant enrichment (*P*<0.05; 1 way ANOVA) in the sample subset that corresponds to their discrete domains of expression in the embryo. Differential expression between regions was determined by the application of a multi step process. Samples were first normalised to the 50th percentile (median) across the entire expression dataset and then each gene was normalised to the median of its own expression across each rhombomere. Thus, gene expression profiles are scaled and centred about 1, where 1 represents the median of a gene’s expression across the experiment. In this way, a gene expression value greater than 1 is classified as enriched whereas a value of less than one is depleted (or absent) in a particular rhombomere with respect to the median level of that gene’s expression across the whole experiment. Error bars represent standard deviation (s.d.) of mean expression levels in each biological replicate.

The relationship within and between biological replicates (m—r5, sets 1-2) was investigated using PCA. ‘Following normalisation (Global to 50th percentile and scaling to median of each gene’s expression level across the whole experiment [m-r5, sets 1 & 2]) and the removal of non expressed genes (i.e., genes defined as absent or marginal [Affymetrix Flag designates] in 2 of 2 biological replicates) and Affymetrix control signal (AFFX controls), the overall distribution of gene expression levels for each sample was examined with PCA (Figure 4; PCA 1=38.3%, PCA 2=12.7%, PCA 3=9.647%). In general, the proximity of the biological replicates (Figure 4; similarly colored dots) to each other reveals that they are more related to each other than any other sample. The clear exception is the r3 biological replicates which are more separated in the PCA space particularly in the PCA 2 & 3 axis.’ To further examine the relationship between replicates, the same set of genes described above were analyzed by Condition Tree using a Pearson Correlation Similarity measure with an Average Linkage clustering algorithm. Using this, biological replicates showed the following distance relationships to each other; M=0.688, r1=0.838, r2=0.908, r3=0.682, r4=0.991, r5=0.871). These data confirm that the r3 biological replicates are the least similar of the experimental set but still within a similar range to the other pairs of replicates. Additionally following statistical processing to identify differentially expressed genes between regions, known markers for each region were correctly assigned to their cognate tissue. For example, Otx2 is significantly enriched (*P*<0.05; 1 way ANOVA) in midbrain microarray samples only which is concordant with its known restricted expression to this tissue in the developing embryo relative to the other samples in this dataset (Figure 5 and Nakamura *et al.*^15^). Similarly, both Fgf8 and Hoxb1 are significantly up regulated (*P*<0.05; 1 way ANOVA) in discrete microarray samples (m/r1 & r4 respectively) in line with the known restricted domains of mRNA expression in an equivalent-stage chick embryo (Figure 5 and refs 4,5,13). This further validates the fidelity of the original dissections and subsequent derived data set. Together these findings demonstrate that the data described here are a faithful representation of gene expression in these regions within the limitations of the array technology.

## Usage Notes

The data provided in this experimental set can be used to determine the set of genes statistically significantly differentially expressed across the developing midbrain and hindbrain of E3 chicken embryos. Using other approaches this cohort of differentially expressed genes can be further refined into groups of genes expressed in each individual tissue (e.g., genes enriched in r4) or combinations of tissues (e.g., genes enriched in r2 & r4). The dataset described here is represented by biological duplicates for each condition. Consequently, this may limit any potential application of this dataset (e.g., pathway analysis) when compared to those with statistical power (e.g., biological triplicates or greater).

### Determination of differentially expressed genes

The data files described here can be analysed with either proprietary (e.g., GeneSpring GX [http://www.genomics.agilent.com]) or freeware packages (e.g., Bioconductor [http://www.bioconductor.org/]) designed for the statistical interrogation and visualisation of microarray-based experiments.

Data can be analysed using the GeneSpring package (Agilent Technologies, Wokingham, Berkshire, UK). Briefly, the suitability of the expression data sets for inclusion in the analysis and the overall relationship between and within the biological replicates is first assessed using quantile plots, principle components analysis (PCA), hierarchical clustering and assessment of internal control performance. Differential gene expression between samples maybe determined by the application of a multi-step process; briefly, samples are first ‘globally’ normalised across the expression dataset to compensate for potential technical variation in the levels of hybridisation of the arrays, and then each gene expression value is scaled to a user defined value (e.g., normalised to the median of a gene signal vale across each rhombomere). Prior to statistical analysis to determine significant differential gene expression between samples, the set of genes to be analyzed can be pre filtered. For example, genes classed as being not expressed (that is, absent [Flag designation] in two of the two biological replicates) or not varying their expression above a user defined level (typically 2 fold) can be removed. Similarly, genes with low ‘raw’ values (Affymetrix signal) across all of the samples (typically raw values between 0.01 and 20 or those genes with expression values in the lowest 20th percentile of the array) can also be removed prior to statistical analysis. From the remaining set of genes, genes whose expression levels differ significantly between each sample can be determined by one-way analysis of variance (ANOVA; *P*=0.05) or other statistical tests. The application of a multiple testing correction is optional. Statistically different genes can be functionally classified using a combination of Gene Ontology (GO) criteria and other molecular descriptions derived from UniGene, GenBank and Entrez Gene databases. Genes may also be clustered into potentially co-regulated groups using both unsupervised and supervised approaches including self-organising maps and quality threshold clustering. A more detailed biological interpretation of the set of differentially expressed genes can be derived from the use of gene network analysis packages (e.g., GeneGO [www.genego.com/] & Ingenuity Systems Pathway Analysis (www.ingenuity.com/).

## Additional information

**How to cite this article:** Wilson, L. & Chambers, D. Transcriptomic analysis of midbrain and individual hindbrain rhombomeres in the chick embryo. *Sci. Data* 1:140014 doi: 10.1038/sdata.2014.14 (2014).

## Supplementary Material



## Figures and Tables

**Table 1:** Affymetrix Cel files (Version 3).

**File name**	**Biological replicate**	**Chick HH11 brain region represented**	**Processing origin**
1_chick_m_set_1.cel	Set 1	Midbrain	Affymetrix GCOS version 1.2
2_chick_r1_set_1.cel	Set 1	Rhombomere 1	Affymetrix GCOS version 1.2
3_chick_r2_set_1.cel	Set 1	Rhombomere 2	Affymetrix GCOS version 1.2
4_chick_r3_set_1.cel	Set 1	Rhombomere 3	Affymetrix GCOS version 1.2
5_chick_r4_set_1.cel	Set 1	Rhombomere 4	Affymetrix GCOS version 1.2
6_chick_r5_set_1.cel	Set 1	Rhombomere 5	Affymetrix GCOS version 1.2
7_chick_m_set_2.cel	Set 2	Midbrain	Affymetrix GCOS version 1.2
8_chick_r1_set_2.cel	Set 2	Rhombomere 1	Affymetrix GCOS version 1.2
9_chick_r2_set_2.cel	Set 2	Rhombomere 2	Affymetrix GCOS version 1.2
10_chick_r3_set_2.cel	Set 2	Rhombomere 3	Affymetrix GCOS version 1.2
11_chick_r4_set_2.cel	Set 2	Rhombomere 4	Affymetrix GCOS version 1.2
12_chick_r5_set_2.cel	Set 2	Rhombomere 5	Affymetrix GCOS version 1.2
The CEL file stores the results of the intensity calculations on the pixel values of the DAT file. This includes an intensity value, standard deviation of the intensity, the number of pixels used to calculate the intensity value, a flag to indicate an outlier as calculated by the algorithm and a user defined flag indicating the feature should be excluded from future analysis. The file stores the previously stated data for each feature on the probe array. The information below describes the version (Affymetrix).			
Version 3 is generated by the MAS software. This was also known as the ASCII version.			

**Table 2:** Affymetrix CHP (Txt) files (MAS5 pre-processed files).

**File name**	**Biological replicate**	**Chick HH11 brain region represented**	**Pre-processing origin**
1_chick_m_set_1.txt	Set 1	Midbrain	GCOS 1.2 MAS5 algorithm
2_chick_r1_set_1.txt	Set 1	Rhombomere 1	GCOS 1.2 MAS5 algorithm
3_chick_r2_set_1.txt	Set 1	Rhombomere 2	GCOS 1.2 MAS5 algorithm
4_chick_r3_set_1.txt	Set 1	Rhombomere 3	GCOS 1.2 MAS5 algorithm
5_chick_r4_set_1.txt	Set 1	Rhombomere 4	GCOS 1.2 MAS5 algorithm
6_chick_r5_set_1.txt	Set 1	Rhombomere 5	GCOS 1.2 MAS5 algorithm
7_chick_m_set_2.txt	Set 2	Midbrain	GCOS 1.2 MAS5 algorithm
8_chick_r1_set_2.txt	Set 2	Rhombomere 1	GCOS 1.2 MAS5 algorithm
9_chick_r2_set_2.txt	Set 2	Rhombomere 2	GCOS 1.2 MAS5 algorithm
10_chick_r3_set_2.txt	Set 2	Rhombomere 3	GCOS 1.2 MAS5 algorithm
11_chick_r4_set_2.cel	Set 2	Rhombomere 4	GCOS 1.2 MAS5 algorithm
12_chick_r5_set_2.cel	Set 2	Rhombomere 5	GCOS 1.2 MAS5 algorithm
The CHP file contains probe set analysis results generated from Affymetrix software. There are several versions of CHP files generated by the MAS5, GCOS and other Affymetrix software. Each line in the text file contains an Affymetrix probe set ID (representing a gene sequence) and a corresponding expression value derived from MAS5 processing of the Cel file. A limited amount of biological annotation about each probe set ID is also included.			
